# Analysis of changes and influencing factors of stablization treatment effects and bioavailability after freeze-thaw: a case study of Pb-contaminated soil in a non-ferrous metal factory in Northeast China

**DOI:** 10.3389/fmicb.2024.1512899

**Published:** 2024-12-17

**Authors:** Wangwang Hao, Dongdong Wang, Miao Yu, Yun Cai, Yu Wang

**Affiliations:** ^1^Key Laboratory of Groundwater Resources and Environment, Ministry of Education, Jilin University, Changchun, China; ^2^Technical Centre for Soil, Agricultural and Rural Ecology and Environment, Ministry of Ecology and Environment, Beijing, China

**Keywords:** Pb, contaminated site, bioaccessibility, stabilization, free-thaw cycles, microbial community

## Abstract

**Introduction:**

Solidification/Stabilization techniques are commonly used for the containment and isolation of Pb-contaminated soil, but they cannot reduce the amount of contaminants. Freeze – thaw after stabilization may affect Pb’s environmental behavior and increase the uncertainty of environmental risk.

**Methods:**

*In vitro* experiments can simulate the bioavailability of heavy metals to the human body, accurately assessing their environmental health risks. In this study, soil samples from Pbcontaminated site are collected from a non-ferrous metal plant in Northeastern China. Through the results of stabilization and freeze–thaw after stabilization experiments, analyzing the changes of physicochemical property, Pb treatment effects (total concentration, leaching concentration, and occurrence forms) and microbial communities, and studying the influencing factors of Pb’s bioavailability.

**Result and discussion:**

The results show that stabilization and freeze – thaw after stabilization directly alter soil physicochemical property, thereby affecting the leaching and occurrence form of Pb and microbial communities, and closely related to changes in bioavailability of Pb. Both stabilization and freeze–thaw treatment reduced the leaching concentration of Pb, decreased the proportion of available Pb (acid-soluble state, oxidation state and reduction state), increased the bioavailability of Pb in the gastric phase, but decreased in the intestinal phase; And the dominant bacterial phylum in the soil changed to Firmicutes, the dominant bacterial genus changed to *Bacillus*; The analysis of the results shows that the bioavailability of Pb is related to soil pH, cation exchange capacity (CEC), soil organic matter (SOM), soil moisture content (SMC), Pb (leaching, acid soluble state, oxidation state, residual state), types of microorganisms in soil.

## 1 Introduction

Due to intensified activities such as mining, smelting, and fossil fuel combustion, Pb has become one of the most widely distributed toxic metals in the world (Cheng and Hu, 2010). The remediation of Pb-contaminated soils has become a challenging and hot topic in the global environmental field. In China, Pb-contaminated farmland accounts for approximately 20% of the total heavy metal contaminated farmland area (Feng et al., 2020). Pb in soil is difficult to decompose and highly toxic, persisting in the environment for long periods. It can accumulate in plants or enter the human body through the food chain, where it binds to various enzymes and interferes with physiological functions. This can severely damage the nervous, digestive, immune, and reproductive systems and may also cause cancer, posing significant health risks (Sage et al., 2023). Solidification/Stabilization techniques are the primary method for remediating Pb-contaminated soils (Kumpiene et al., 2008; Zhang T. et al., 2020; Zada et al., 2023). By adding stabilizers to the contaminated soil, chemical reactions (such as adsorption, precipitation, ion exchange, etc.) occur with heavy metals like Pb, altering their forms and thus reducing their mobility, bioaccessibility, and leaching toxicity (Jiang et al., 2022). Stabilization techniques offer advantages such as short treatment times and a wide range of applicability. However, they can’not reduce the total amount of contaminants. Therefore, their long-term effectiveness is difficult to ensure. Changes in environmental conditions such as soil pH, humidity, and temperature might re-mobilize the Pb, threaten human health again (Fu and Lu, 2019; Li et al., 2021).

Permanent and seasonal frozen soils account for 23% of the global land area. The freeze–thaw (F&T) action of the surface soil in these regions affects the environmental behavior of heavy metals and increases the uncertainty of risk. However, the impact of F&T action on the effectiveness of soil stabilization and remediation is still unclear (Zhou et al., 2018; Okereafor et al., 2020). Studies have shown that F&T cyclic can enhance the stabilization effect of Cu, Zn and Cd by changing soil physicochemical properties (Hou et al., 2020; Wang et al., 2021). Other studies have indicated that F&T action can alter the adsorption and available forms of Zn, Pb, and Cd in the soil, causing them to be reactivated (Yang et al., 2022). These heavy metals can enter the human body through ingestion, skin contact, and inhalation of particulate matter. Bioaccessibility refers to the extent to which pollutants can be absorbed by the human body. Using *in vitro* experiments to simulate the process of heavy metal contaminations entering the human gastrointestinal tract through oral and respiratory pathways can indicate the risk of contaminations to human health (Xu et al., 2022). The United States has included bioaccessibility as an indicator in the assessment of health risks from heavy metal contaminated soil (United States Environmental Protection Agency, 2007). Exploring the key factors affecting the bioaccessibility of heavy metals such as Pb in soil has become a hot research topic (Sun et al., 2023). However, current research is mainly limited to the study of the impact of single factors on bioaccessibility, such as different methods *in vitro* simulation, soil physicochemical properties (pH, particle size, organic matter content, etc.), heavy metal leaching concentration and the change of occurrence morphology (Bradham et al., 2018; Griggs et al., 2021; Yin et al., 2021). There is still a lack of analysis on the relationship between microbial communities and changes in bioaccessibility and a comprehensive analysis of factors affecting bioaccessibility. Microorganisms can directly or indirectly affect the solubility and occurrence morphology of heavy metals by changing soil pH, producing organic matter, and adsorbing heavy metals, thereby altering heavy metals’ bioaccessibility (Yin et al., 2021). Therefore, microorganisms should be a key consideration when analyzing changes in the bioaccessibility of heavy metals.

Based on the above investigation, this study collects soil samples from a non-ferrous metal smelter in a seasonal permafrost region of Northeast China. Intended to simulate stabilization and subsequent long-term freeze–thaw cycles to compares the physicochemical properties, leaching toxicity of Pb, occurrence forms, bioaccessibility, and the types and quantities of microorganisms in the soil under three conditions: the original soil (Step1), stabilized soil (Step2), and stabilized soil after F&T cycles (Step3). The study aims to investigate the following questions: (1) The impact of F&T cycles on the physicochemical properties of Pb-contaminated soil after Step2; (2) The impact of F&T cycles on the migration and transformation of Pb in the soil after Step2; (3) Changes in dominant microbial composition and structure in Pb-contaminated soil after Step2 and Step3; (4) Interpret the interactive influences among soil physicochemical properties, Pb’occurrence morphology, dominant microbial species, and Pb’s bioaccessibility.

## 2 Materials and methods

### 2.1 Study site and sample collection

The sampling location is at a non-ferrous metal smelter in Northeast China. This facility integrates non-ferrous metal smelting with chemical production, involving four main production systems for Zn, Pb and Cu along with associated comprehensive utilization and waste heat power generation systems. The zinc smelting systems use vertical retort smelting, hydrometallurgical electrolysis, and sealed blast furnace processes, respectively. The copper refining system adopts the small plate electrolytic refining processes. During the production process, Pb and other heavy metals can be released into the surrounding environment through wastewater, exhaust gases, and solid waste, radiating outward from the factory site. Some studies have shown that the Pb’s concentration in some spots of the surface soil (0–20 cm) in nearby villages reaches 55 mg/kg (Chang et al., 2017).

**Figure 1:**
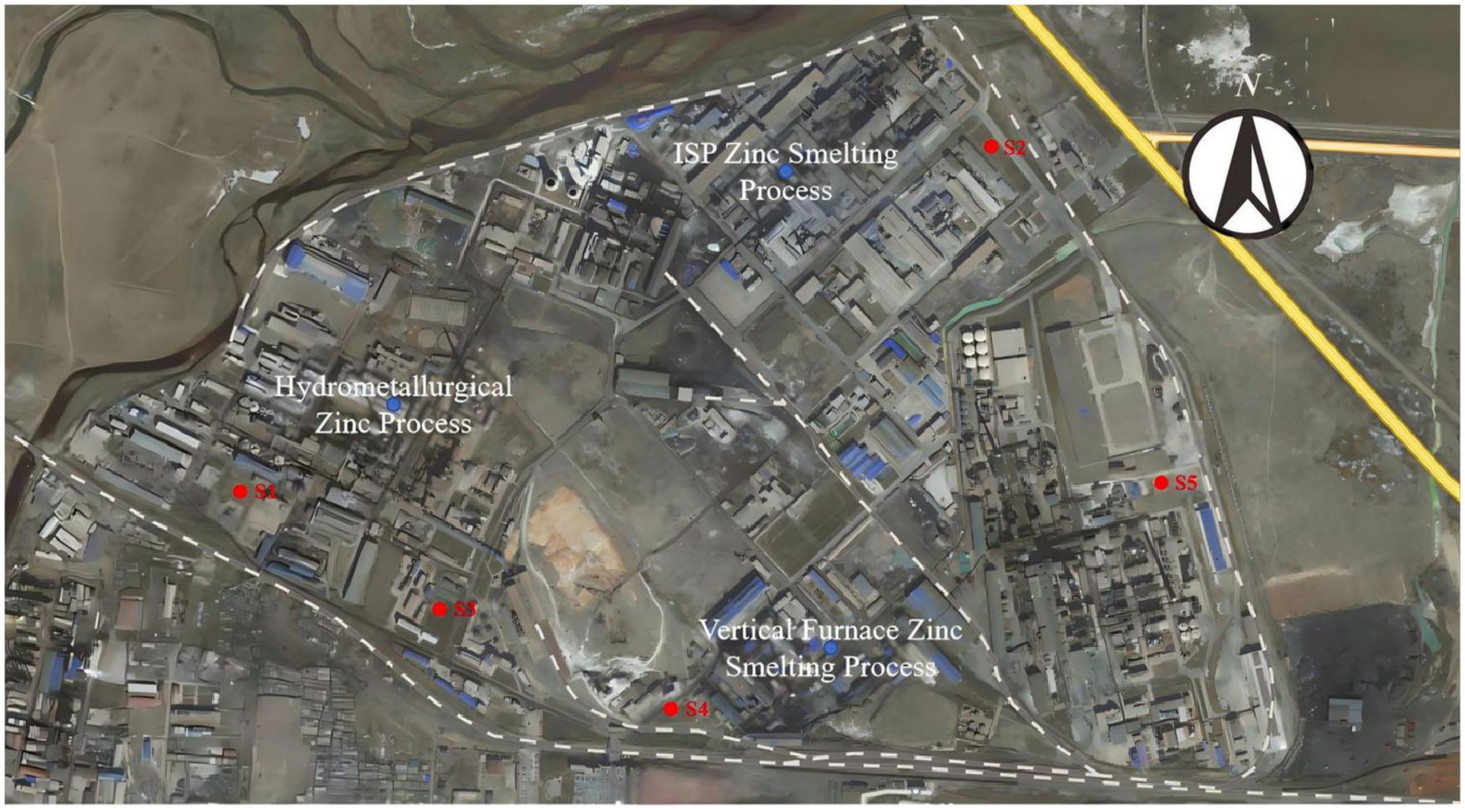
The sampling locations S1 ~ S5.

Set up soil sampling points near the metal smelting area, and collect five samples from the sites with higher concentrations of heavy metals selected by rapid detection with XRF (Figure 1). S1 and S3 are located to the southwest and southeast of the hydrometallurgical zinc smelting area, respectively; S2 is close to the east side of the ISP blast furnace zinc smelting process system; S4 is situated to the southwest of the vertical retort zinc smelting area; S5 is at the farthest eastern point of the factory premises. Collect the surface soil (0–20 cm), and air-dry it naturally after removing stones, twigs, dead leaves and other debris. The dried samples were sieved through a 10-mesh sieve to separate materials larger than 2 mm, such as sand and gravel. One portion of the sieved soil sample was placed into sealed bags for storage and future testing, while another portion was stored at −80°C in a low-temperature refrigerator for microbial sequencing analysis.

### 2.2 The experiment of stabilization and freeze-thaw

#### 2.2.1 Stabilization experiment

Weigh 500 g soil sample and 1 g of FeSO_4_ and 5 g of CaO were added as stabilizers. Deionized water was added to create slurries at a liquid-to-solid ratio of 1.2:1 (L/kg), then stir well and let stand (Fang et al., 2023). During the stabilization period, the water curing time should be more than 7 days (Ministry of Ecology and Environmental of the People’s Republic of China, 2023). To ensure the agent has sufficient time to react with contaminations, this study placed the soil sample indoors and stabilized it under natural conditions for 8 days. Weigh the culture bottle every 3 days and replenish the soil with water to maintain a moisture content of 50%.

#### 2.2.2 Stabilization followed by freeze-thaw experiments

Weigh 250 g of the stabilized soil sample mentioned above and place it in a polyethylene plastic white bottle. Considering the climate conditions in Northeast China, soil samples were frozen at −15°C for 12 h and melted at 10°C for 12 h (Ministry of Transport of the People’s Repulic of China, 2020). One cycle of freezing and melting is considered as one cycle (Juan et al., 2018). Set 10 cycles and maintain a moisture content of 50% during the F&T.

### 2.3 Determination of soil physicochemical properties

The determination includes pH, electrical conductivity (EC), soil moisture content (SMC), cation exchange capacity (CEC), and soil organic matter content (SOM). The pH and EC values were measured using the electrode method, the CEC was determined by the hexamminecobat trichloride solution-spectrophometric method, the SOM was measured by the potassium dichromate titration method and the SMC was determined by the oven-drying method.

### 2.4 Determination of heavy metal leaching concentration and occurrence forms

Determination of concentration of Pb’s leachate. Referenced the horizontal shaking method (Ministry of Ecology and Environmental of the People’s Republic of China, 2010). Added water to the extraction bottle at a liquid-to-solid ratio of 10:1 (L/kg), tightly closed the bottle cap, and securely fixed it vertically on a horizontal shaking device. The shaking frequency was 110 ± 10 times/min, with an amplitude of 40 mm. After shaking for 8 h at room temperature, removed the extraction bottle and let it stand for 16 h. Then, measured the concentration of heavy metals in the leachate.

Determination of concentration of Pb’s occurrence forms. Refer to the BCR three-step method (Cappuyns et al., 2007): specific operations are described in Table 1.

**Table 1:** Determination of the heavy metal speciation in soil by BCR method.

Step	Speciation	Extractants	Experimental conditions
1	Acid-soluble	0.11 mol/L HAc	Shake at room temperature for 16 h, centrifuge at 3000 r/min for 20 min
2	Reduced state	0.50 mol/L NH_2_OḤHCI	Shake at room temperature for 16 h, centrifuge at 3000 r/min for 20 min
3	Oxidation state	8.8 mol/L H_2_O_2_	Shake at room temperature for 1 h and heat to 85 ± 2°C for 1 h
		8.8 mol/L H_2_O_2_	Heat at 85 ± 2°C for 1 h
		1 mol/L(pH = 2)NH_4_OAc	Shake at room temperature for 16 h
4	Residue state	70%HNO_3_ + 60%HCLO_4_ + 40%HF	75°C for 3 h, 90°C for 2 h, 125°C for 3 h, 170°C for 5 h, 190°C for all dry, the residue was digested at 10 mL of 5% HNO_3_ at 75°C for 1 h

### 2.5 The bioaccessibility assessment of Pb

The use of *in vitro* gastrointestinal experiments to simulate the extraction of heavy metals by digestive fluids is a strong indicator of bioaccessibility (Wu et al., 2015). The composition of the simulated gastrointestinal fluid were prepared according to the components of the gastrointestinal fluid for the Chinese population recorded in the Chinese Pharmacopoeia (Lei et al., 2020). This study used the SBRC method to determine the concentration of Pb in digestive fluids. A portion of each soil sample was passed through a 0.25 mm nylon sieve for bioaccessibility testing. The specific operation steps are as follows:

1. Gastric phase. adjust the pH to 1.5 using a 12 mol/L concentrated HCl solution (freshly prepared and used), preheat to 37°C for 2 h, and then add 30 mL of simulated gastric juice. Then place it in the constant temperature water bath shaker (37°C and 150 r/min) to shake for 1 h, while keeping it constant with a 12 mol/L concentrated HCl solution and NaHCO_3_ powder. Finally, centrifuge the digestion solution at 3000 r/min for 15 min, take 10 mL of sample using a disposable syringe, filter it through a 0.45 μm membrane, and store it at 4°C for testing.
2. Intestinal phase. preheat the prepared intestinal fluid to 37°C 2 h before use. After gastric digestion, adjust the pH to 7.0 using a 12 mol/L concentrated HCl solution and NaHCO_3_ powder, and add it to intestinal fluid. Continue oscillating for 4 h under the same conditions and maintain a constant concentration of 12 mol/L HCl solution and NaHCO_3_ powder. Finally, centrifuge the digestion solution at 3000 r/min for 15 min, take 10 mL of sample using a disposable syringe, filter it through a 0.45 μm membrane, and store it at 4°C for testing.

The method of the bioaccessibility and the bioaccessibility concentration of Pb in soil during the gastrointestinal digestion phase are as follows:


$$\begin{array}{l} C_{g}=\frac{\rho_{g}\times v_{g}}{m}\\ \mathrm{BAG}=\frac{C_{g}}{C_{s}}\times 100\%\\ C_{gi}=\frac{\rho_{gi}\times v_{gi}}{m}\\ BAI=\frac{C_{gi}}{C_{s}}\times 100\% \end{array}$$

where *C_g_* is the human acceptable concentration of heavy metals in the gastric digestion stage (mg/kg); *C_gi_* is the human acceptable concentration of heavy metals in the intestinal digestion stage (mg/kg); *ρ_g_* is the concentration of heavy metals in simulated gastric juice (ug/L); *ρ_gi_* is the concentration of heavy metals in simulated intestinal fluid (ug/L); *v_g_* is the volume of simulated gastric fluid (L); *v*_*g*i_ is the simulated intestinal fluid volume (L); m is the total amount of soil samples during the test (g); *C_S_* is the concentration of heavy metals in soil samples (mg/kg); *BAG* is the human bioaccessibility of heavy metals in gastric digestion phase, %; *BAI* is the human bioaccessibility of heavy metals in intestinal digestion phase, %.

### 2.6 Microbial sequencing

16S rRNA high-throughput sequencing analysis is conducted as follows: DNA was extracted from fresh soil using the E.Z.N.A.® Soil DNA Kit (Omega Bio-tek, Norcross, GA, USA). The quality of the extracted DNA was checked by 1% agarose gel electrophoresis, and the concentration and purity of the DNA were determined using a micro-ultraviolet spectrophotometer. The V3-V4 region of the 16S rRNA gene was amplified using specific primers. The PCR products were sequenced using the Illumina MiSeq platform.

### 2.7 Data statistical analysis

#### 2.7.1 Soil physicochemical property, occurrence forms of Pb, leaching concentration, and total concentration data analysis

Using Microsoft Office Excel 2020 software to perform data statistics and calculate the percentage changes of variables in Step1, Step2 and Step3, and use Origin 2021 software to draw graphs to visually reflect their changes.


$$W=\left(\frac{p_{1}-p_{2}}{p_{1}}\right)\times 100\%$$

Where *W* is the percentage changes of variables, %; *p_1_* is the compared value; *p_2_* is the comparison value.

#### 2.7.2 Analysis of changes in microbial community structure

Utilize the Major Bioinformatics Analysis Cloud Platform for microbiological information analysis. The Coverage index can reflect the coverage of sequences in a sample, and this value was used to test whether the sequencing results can represent the true situation of microorganisms in the sample. The Chao1 index is used to estimate species richness, and a higher value indicates a richer variety of species in the sample. And use the Shannon index to reflect the alpha diversity of the community and estimate the trend of changes in microbial diversity in the sample.


$$\mathrm{Coverage}=1-\frac{n_{1}}{N}$$

Where *Coverage* is the coverage rate of sampling, %; *n_1_* is the number of species that only appear once in the sample; *N* is the total number of sequences appearing in the samples.


$$\mathrm{Chao}1=S_{obs}+\frac{n_{1}\left(n_{1}-1\right)}{2\left(n_{2}+1\right)}$$

Where *S_obs_* is the number of species observed in the sample; *n_1_* is the number of species that only appear once in the sample; *n_2_* is the number of species that appear twice in the sample.


$$\mathrm{Shannon}=-\overset{\mathrm{Sobs}}{\underset{i=1}{\sum }}\frac{n_{i}}{N}\ln\frac{n_{i}}{N}$$

Where *n_i_* is the all sequences contained in the *i*-th sample. N is the total number of sequences appearing in the sample.

#### 2.7.3 Correlation analysis

Pearson correlation analysis was conducted using Origin 2021 software to analyze linear relationships between different variables. The closer the absolute value of the correlation coefficient *r* is to 1, the stronger the correlation. When the significance coefficient *p* ≤ 0.05, the correlation between the two variables is statistically significant.

#### 2.7.4 Geographical detector

Geographical Detector is an emerging statistical method primarily used to test whether variables have a significant impact on the spatial heterogeneity of a phenomenon. If the independent variable has a significant impact on the dependent variable, their change trends should exhibit similar distribution characteristics (Wang and Xu, 2017). This research employs the Geographical Detector model to interpret the explanatory power of variables that are correlated with the bioaccessibility of Pb in the gastric and intestinal stages on their change trends. The core of the Geographical Detector is to assess the explanatory power of independent variables on the spatial heterogeneity of the dependent variable through the calculation of *q* values.


$$q=1-\frac{\sum_{h=1}^{L}M_{h}\sigma_{h}^{2}}{M\sigma^{2}}$$

Where *M* is the total number of samples; *M_h_* is the number of samples in the *h*-th sub-region; *σ* is the overall variance; *σ_h_* is the variance in the *h*-th sub-region; *L* is the number of sub-regions.

## 3 The result and discussion

### 3.1 The effects of stabilization and post-stabilization F&T on the physicochemical properties of soil

The properties of the original soil underwent the following changes after Step2 and Step3 (Table 2). (1) pH. The addition of stabilizing agents FeSO_4_ and CaO increased the average pH of the soil from 7.4 to 9.8. During the F&T cycle, as water froze and evaporated, salts became increasingly concentrated. The high salt concentration suppresses the activity of acidic substances, causing the average pH of the soil to further increase to 10.4; (2) SMC. During the stabilization process, continuous water supplementation caused the SMC to increase by 1.36 to 58.52% with the addition of stabilizing agents. In contrast, the F&T cycles led to repeated compression and closure of soil pore structures, reducing the soil porosity and water-holding capacity, resulting in a decrease in SMC by 88.21 to 92.29%; (3) SOM. The stabilizing agents converted the SOM into more stable and less decomposable forms, resulting in a decrease in SOM by 30.11 to 45.24%. F&T cycles, on the other hand, promoted the mineralization of SOM by soil microorganisms (Juan et al., 2018), leading to a further decrease in SOM by 32.25 to 49.86%; (4) EC and CEC. Step2 and Step3 processes enhanced the competition of strongly soluble salts such as Na^+^ and K^+^ in the soil, increasing the number of negatively charged on the soil surface. This resulted in higher values of EC and CEC.

**Table 2:** Soil physicochemical properties of Step1, Step2 and Step3.

Sample		pH	EC (us/cm)	SMC (%)	CEC (cmol/kg)	SOM (%)
Step1	S1	7.43	126	13.11	22.03	4.96
	S2	7.40	108	11.62	18.28	5.13
	S3	7.43	83	12.93	12.18	5.04
	S4	7.42	113	13.97	17.93	5.28
	S5	7.44	101	12.58	12.94	5.01
Step2	S1	9.51	120 (−4.76%)	15.15 (15.56%)	347.73 (1478.44%)	2.72 (−45.16%)
	S2	9.15	144 (33.33%)	18.42 (58.52%)	391.46 (2041.47%)	3.00 (−41.52%)
	S3	10.09	127 (53.01%)	17.50 (35.34%)	383.77 (3050.82%)	2.76 (−45.24%)
	S4	9.24	114 (0.88%)	14.16 (1.36%)	364.40 (1932.35%)	3.69 (−30.11%)
	S5	11.07	210 (107.92%)	13.75 (9.30%)	395.84 (2959.04%)	3.02 (−39.72%)
Step3	S1	9.78	118 (−1.67%)	1.76 (−88.38%)	338.09 (−2.77%)	1.56 (−42.65%)
	S2	10.00	128 (−11.11%)	2.00 (−89.14%)	393.78 (0.59%)	1.79 (−40.33%)
	S3	11.05	144 (13.39%)	1.35 (−92.29%)	266.08 (−30.67%)	1.87 (−32.25%)
	S4	9.96	123 (7.89%)	1.67 (−88.21%)	324.11 (−11.06%)	1.85 (−49.86%)
	S5	11.06	184 (−12.38%)	1.27 (−90.76%)	272.42 (−31.18%)	1.64 (−45.70%)

### 3.2 The effects of stabilization and post-stabilization F&T on the Pb’s migration and transformation of the soil

The toxicity and environmental behavior of heavy metals are closely related to their total concentration, leaching concentration, occurrence forms, specific surface area and particle size distribution (Chuai et al., 2022). Among these, the occurrence forms is the decisive factor for their environmental leaching and mobility (Król et al., 2020). To investigate the effects of stabilization and F&T processes on the environmental behavior of Pb, the total concentration, leaching concentration, and occurrence forms of Pb are measured in Step1, Step2 and Step3 (Table 3).

**Table 3:** Total concentration, leaching concentration and each occurrence form concentrationof Pb in soil.

Sample		Leachable concentration of Pb (ug/L)	Total concentration of Pb (mg/kg)	Acid-soluble state (mg/kg)	Oxidation state (mg/kg)	Reduction state (mg/kg)	Residual form (mg/kg)
Step1	S1	3644.09	533.17	105.54	153.04	250.34	24.22
	S2	3542.60	752.66	121.11	179.30	362.98	89.25
	S3	3438.09	555.13	110.00	153.28	237.32	54.51
	S4	3585.38	770.07	121.67	165.82	365.46	117.11
	S5	3534.34	533.13	108.69	24.14	93.23	25.76
Step2	S1	311.08 (91.46%)	199.14 (62.65%)	16.59 (84.28%)	19.61 (87.19%)	128.28 (48.76%)	34.64 (−43.02%)
	S2	291.95 (91.76%)	311.76 (58.58%)	19.63 (83.79%)	24.17 (86.52%)	155.78 (57.08%)	112.16 (−25.67%)
	S3	192.33 (94.41%)	335.38 (39.59%)	27.27 (75.21%)	28.03 (81.71%)	148.68 (37.35%)	131.40 (−141.06%)
	S4	174.18 (95.14%)	269.22 (65.04%)	28.76 (76.36%)	32.54 (80.38%)	173.13 (52.63%)	34.78 (70.30%)
	S5	231.09 (93.46%)	137.57 (74.20%)	21.12 (80.57%)	23.38 (3.15%)	92.43 (0.86%)	0.62 (97.59%)
Step3	S1	—	146.85 (72.46%)	1.03 (99.02%)	2.84 (98.14%)	56.12 (77.58%)	86.85 (−258.59%)
	S2	—	167.06 (77.80%)	1.03 (99.15%)	0.00 (100%)	68.28 (81.19%)	98.43 (−10.29%)
	S3	—	279.76 (49.60%)	6.58 (94.02%)	4.16 (97.29%)	115.30 (51.42%)	153.70 (−181.97%)
	S4	—	247.24 (67.89%)	6.92 (94.31%)	2.77 (98.33%)	95.27 (73.93%)	142.27 (−21.48%)
	S5	—	101.82 (80.90%)	0.00 (100%)	0.00 (100%)	19.42 (79.17%)	84.82 (−229.27%)

**Figure 2:**
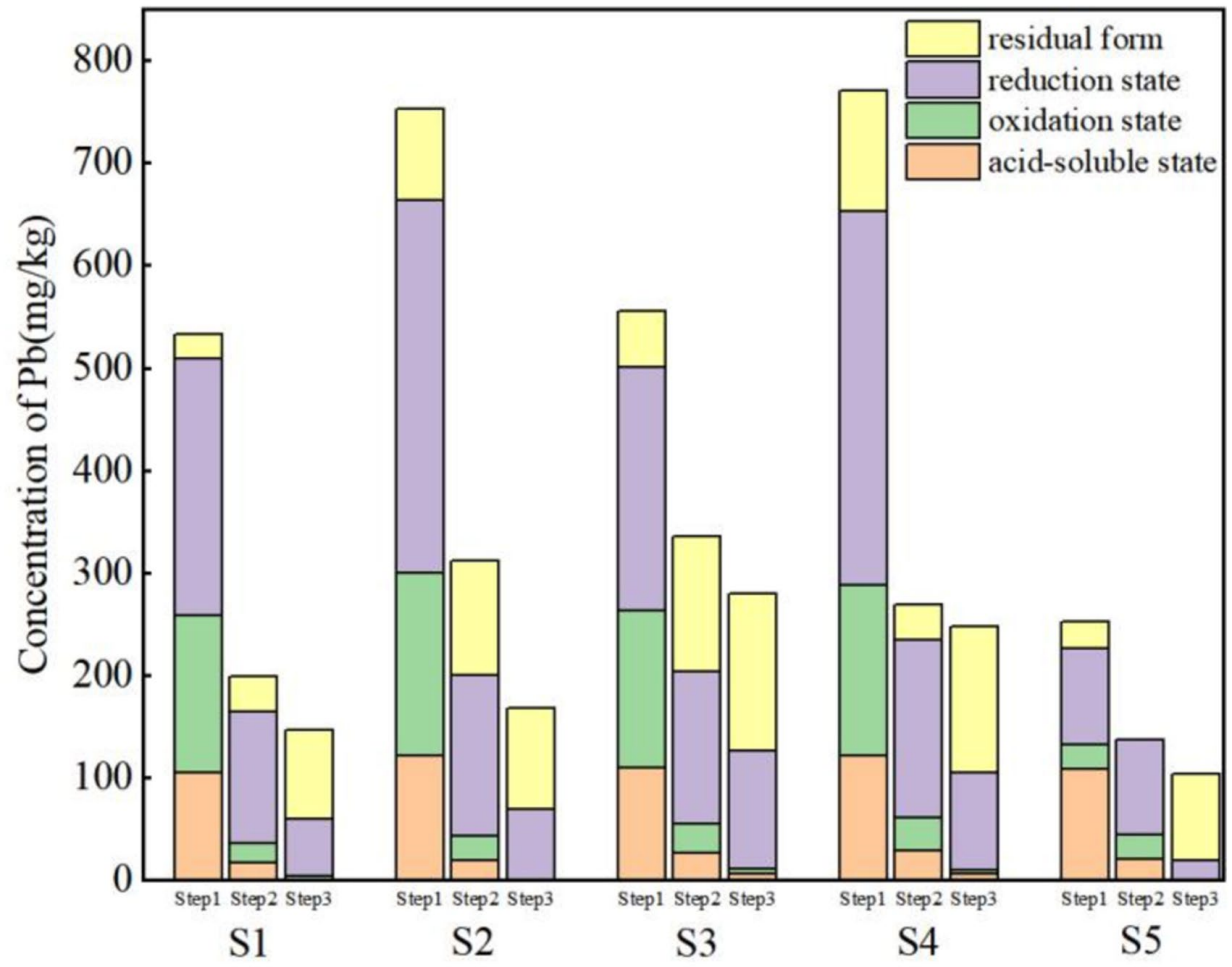
Occurrence of Pb in Step1, Step2 and Step3.

**Figure 3:**
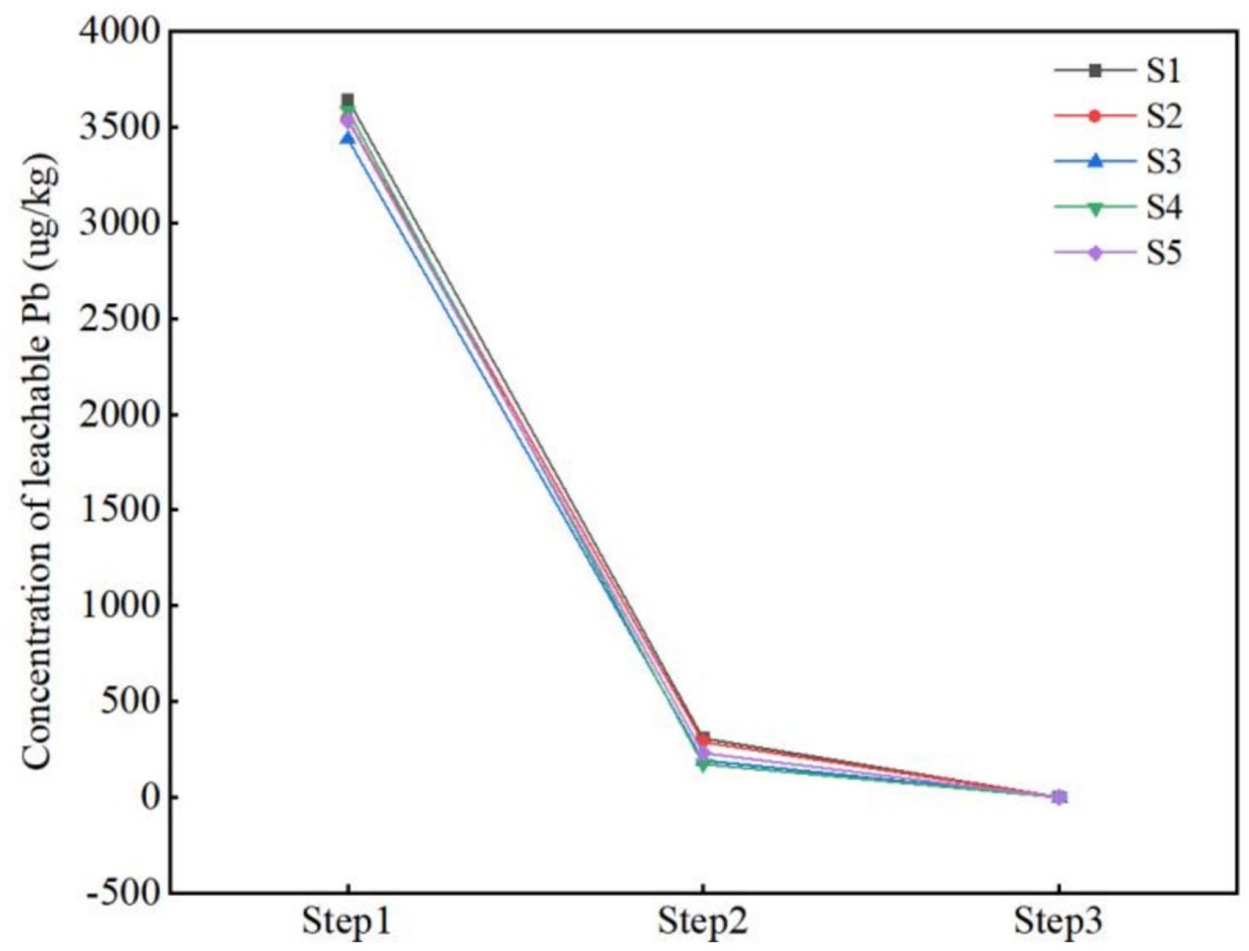
Change in Pb’s leaching concentration.

Original soil had a high total Pb concentration, with a significant proportion of available forms (acid-soluble, reducible, and oxidizable fractions) (Figure 2). The Pb concentrations of five original soil ranged from 3238.09 to 3644.09 ug/L (Figure 3), which were below the Pb leaching concentration threshold (Ministry of Ecology and Environmental of the People’s Republic of China, 2007). However, the total concentrations of Pb in the soil were 533.13 to 770.07 mg/kg, potentially posing a hidden danger to human health. According to the experimental results, Pb had strong availability and mobility in the environment, with the available fractions of Pb at sites S1 to S5 accounting for 95.46, 88.14, 90.18, 84.79, and 89.77%, respectively. Compared with other forms, Pb has the highest proportion of residual form (42.5% ± 6%). If the redox conditions or pH in the environment change, a large amount of Pb may be released (Huu Hieu et al., 2012; Hindersmann and Mansfeldt, 2014).

After Step2, the mobility and availability of Pb were effectively reduced. The highest removal rate of Pb at the S5 site was 74.19%, and the concentration of Pb at each site was significantly reduced, all of which reached the standard of Class I land use (Ministry of Ecology and Environmental of the People’s Republic of China, 2018). During the stabilization process, CaO combined with Pb to form hydroxide precipitate (Zhang P. et al., 2020), and FeSO_4_ combined with Pb to form sulfide precipitate (Ma et al., 2019), so the leaching concentration of Pb decreased by 91.46% ~ 95.14%, the proportion of available state at S2 and S3 sites decreased to 64.02 and 60.82%, and the available state concentrations of S1, S4 and S5 decreased by 344.44 mg/kg, 418.52 mg/kg and 89.13 mg/kg, respectively, compared with Step1, Step3 promotes the Step2’s effect and further reduced the mobility and availability of Pb.

Step3 promoted the Step2’s effect and further reduced the mobility and availability of Pb. After Step3, the concentrations of Pb leaching solution were below the detection limit. The removal rate of Pb in soil increased by 9.8, 19.2, 10.01, 2.85 and 6.7%, respectively, and effective state Pb at S1 ~ S5 decreased to 40.85, 41.32, 45.05, 42.45 and 18.63%. F&T can lead to the destruction of soil aggregates, increase the looseness of the soil, and thus increase the contact surface area and adsorption effect of Pb with the soil. In addition, the pH of each point increased to different degrees after F&T, and the net negative charge on the soil surface increased, resulting in an increase in the adsorption sites of heavy metal ions, thereby increasing the adsorption capacity of Pb. On the other hand, adsorption requires exothermic retention, and the freezing process accelerates the adsorption process of Pb in the soil due to the decrease in temperature.

### 3.3 The effects of stabilization and post-stabilization F&T on the Pb’s bioaccessibility of the soil

**Figure 4:**
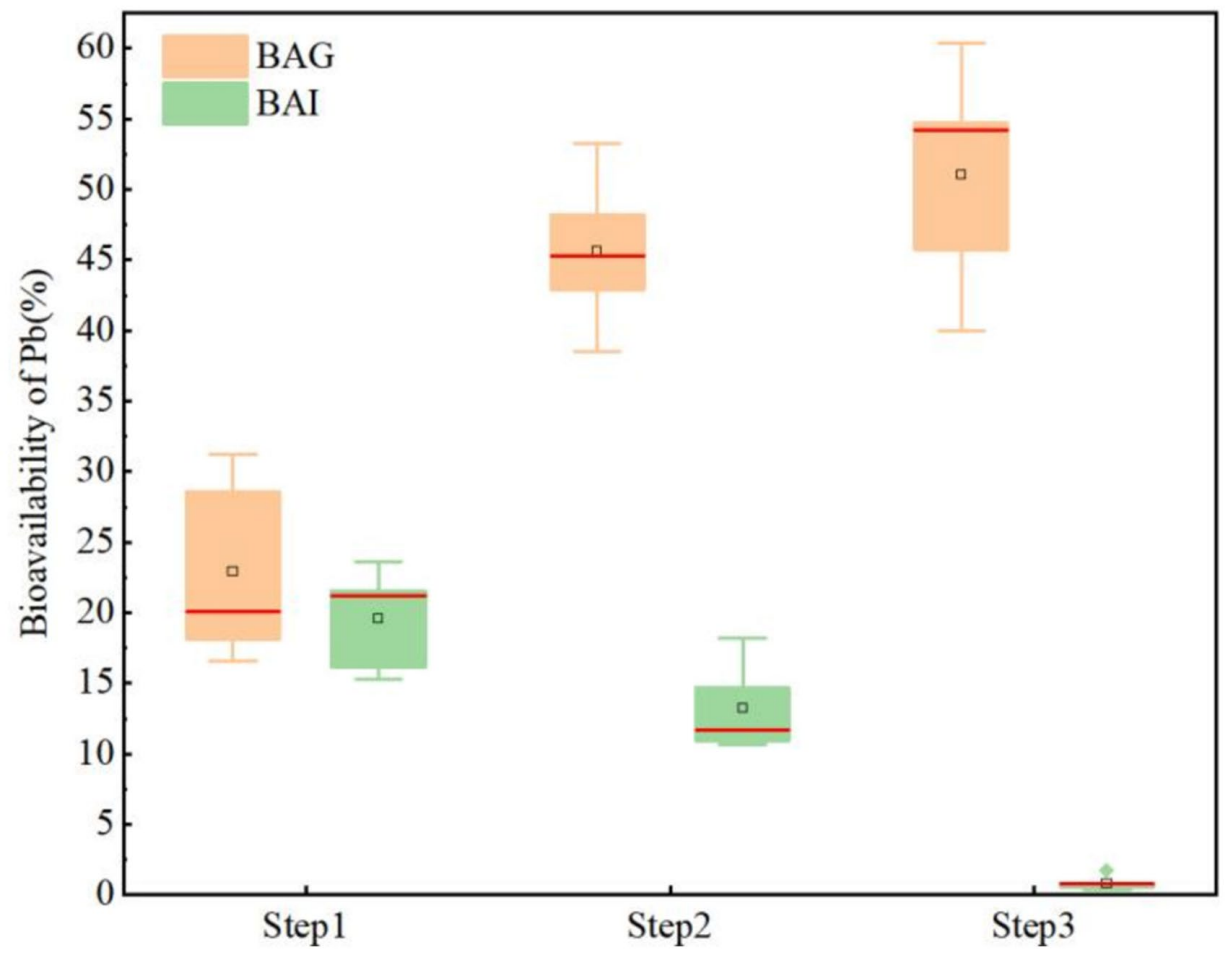
Changes in lead bioaccessibility after Step2 and Step3.

Heavy metals in soil, after biological accumulation into the food chain, pose significant potential hazards to human health. Bioaccessibility is a key factor in determining the health risks of contaminations and in developing effective remediation strategies (Lu et al., 2021). According to the experimental results, the bioaccessibility of the stomach stage in the soil increased continuously, but the intestinal stage was reversed, and the overall percentage of bioaccessibility increased after Step2 and Step3 in the soil (Figure 4). The average bioaccessibility of Pb in Step1 is 22.91% (16.54% ~ 31.24%) in the stomach stage and 19.57% (15.31% ~ 23.65%) in the intestinal stage. And the average bioaccessibility in the stomach stage increased to 45.64% (38.56% ~ 53.29%) in Step2, and decreased to 13.24% (10.64% ~ 18.24%) in the intestinal stage. After the F&T cycle, the average bioaccessibility continued to increase to 51.01% (39.95% ~ 60.40%) in the gastric stage and 0.84% (0.35% ~ 1.72%) in the intestinal stage. Compared with the soil samples at the three stages, the total bioavailability of Pb of gastrointestinal stages in Step2 and Step3 increased by 8.2 and 4.69%, respectively. Overall, the gastric stage is more bio-giving than the intestinal stage, mainly because the gastric digestion stage is a strong acid environment but the intestinal digestion stage is close to a neutral environment, and the gastric juice at lower pH contains more H^+^, which will occupy more adsorption sites on the soil surface, thereby increasing the content of heavy metals in the gastric juice (Juhasz et al., 2009; Denys et al., 2012). In addition, during the intestinal digestion phase, dissolved heavy metal ions may be re-adsorbed by the soil-like matrix, resulting in a further reduction in bioaccessibility in the intestinal phase (Smith et al., 2011). Based on the significant changes in soil physicochemical properties before and after Step2 and Step3, it is inferred that the changes in the bioaccessibility of Pb in the intestinal and gastric stages at different stabilization stages may be indirectly caused by the changes in soil physicochemical properties, and further analysis of the factors causing the changes in biosibability is needed.

### 3.4 The effects of stabilization and post-stabilization F&T on the microbial communities of the soil

The microbial community in the soil is most directly affected by heavy metal contaminations, and the environmental behavior of heavy metals can cause changes in microbial abundance and dominant microflora (Kuperman and Carreiro, 1997; Li et al., 2017). The results show that the effective sequence coverage rate of each sample is higher than 98% (Table 4), indicating that the sequencing depth is sufficient, and the sequencing results can truly represent the microbial situation in the sample. Among all the points, the microbial community at the S2 was the most diverse and stable while the microbial community at the S1 was the least stable. Compared with the original contaminated soil, the Chao1 index showed a downward trend, the species richness decreased, and the Shannon index also showed a decreasing trend after Step2 and Step3, it can be argued reduced microbial diversity in soils.

**Table 4:** α diversity index of microbial community.

Sample		Chao1	Shannon	Coverage
Step1	S1	3269.70	6.26	98%
	S2	3920.81	6.57	98%
	S3	3256.73	6.37	98%
	S4	3628.99	6.50	98%
	S5	3710.15	6.40	98%
Step2	S1	2334.67	5.17	99%
	S2	3297.37	5.42	98%
	S3	3022.55	6.11	98%
	S4	2502.48	5.06	99%
	S5	2948.19	5.74	98%
Step3	S1	2397.12	5.46	99%
	S2	3131.42	5.57	98%
	S3	3104.91	6.10	98%
	S4	2377.58	5.42	99%
	S5	2788.40	5.53	98%

**Figure 5:**
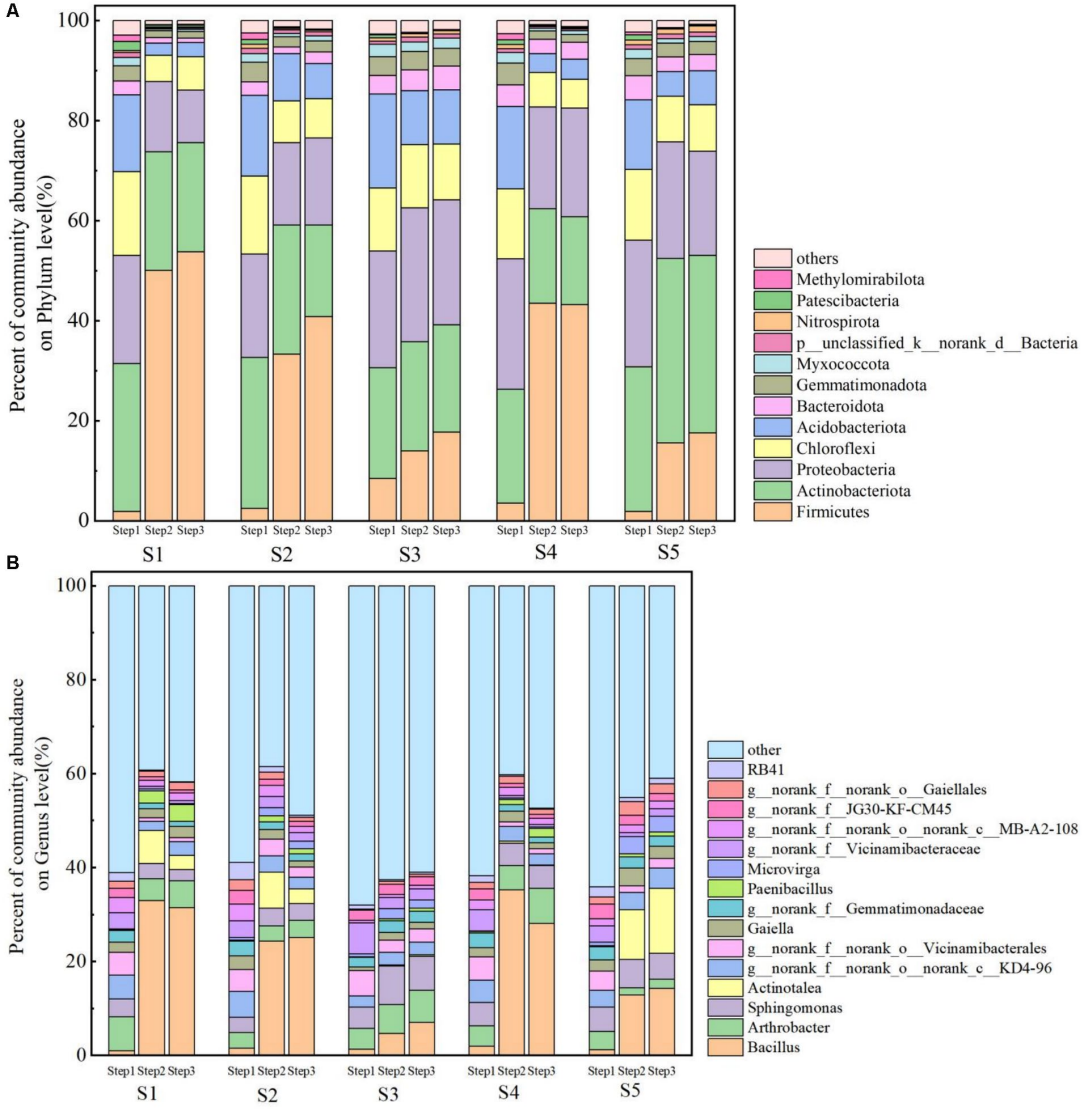
Dominant microbial communities at the phylum level **(A)** and genus level **(B)**.

Sequencing results showed that the microbial community responded to changes in heavy metal contamination levels through selective growth, and that Pb’s contamination levels and environmental conditions had no effect on community richness and composition, but significantly affected microbial community structure (Azarbad et al., 2015; Müller et al., 2021). From phylum and genus level analysis, the top 10 microorganisms with the highest abundance at three treatment stages at the S1–S5 site are selected to draw community histograms (Figure 5). The results showed that the microbial dominant phyla in Step1 are consistent, the sum of the number of microorganisms in Acidobacteriota, Actinobacteriota, Actinobacteriota and Proteobacteria accounted for more than 80% of the total number of microorganisms. *Arthrobacter* and *sphingomonas* are the dominant strains in Step1, belonging to Actinobacteriota and Proteobacteria respectively, which have strong metabolic degradation ability. Some studies had also shown that these species have strong tolerance to heavy metal contaminated soil and have great application value in the field of environmental remediation (Azarbad et al., 2015; Lur et al., 2015). Actinobacteriota, Proteobacteria, Acidobacteriota and Chloroflexi still have a large proportion in Step2 and Step3. However, it is worth noting that the proportion of Firmicutes has significantly increased. After stabilization, the proportion of Firmicutes in S1 site was the highest at 50.01%, and increased to 53.76% after F&T. The proportion of *Actinotalae* increased after stabilization and F&T treatment, but not significantly, such as the proportion at S3 and S4 sites did not increase. On the contary, *Bacillus* is the most dominant genus of bacteria in each site after soil treatment, with the S4 site accounting for up to 35%. *Bacillus* belongs to Firmicutes, which can form spores for dormancy under extreme conditions, has strong resistance to adverse environments and can still survive stably at −20°C (Nagai, 2019). Although the F&T cycle can reduce the swimming activity of bacteria and the transcription of flagellin-coding genes, it can still normally secrete surface pigments to form a stable biofilm and maintain the integrity and original growth rate of cells (Asadishad et al., 2018). Some studies had also shown that spores, biological enzymes, and biopolymers produced by *Bacillus* can be used as additives to improve the effectiveness of soil stabilization treatment (Lin et al., 2019; Ramdas et al., 2021). The tolerance of *Bacillus* to extreme conditions and its characteristics as a bio-based stabilizer can provide new ideas for the solidification/stabilization of contamination soil in permafrost regions, such as the Northeast China.

### 3.5 Correlation analysis between soil physicochemical properties, occurrence forms of Pb, dominant microbial species and bioaccessibility

#### 3.5.1 Correlation analysis between soil physicochemical properties and Pb’s concentration

**Figure 6:**
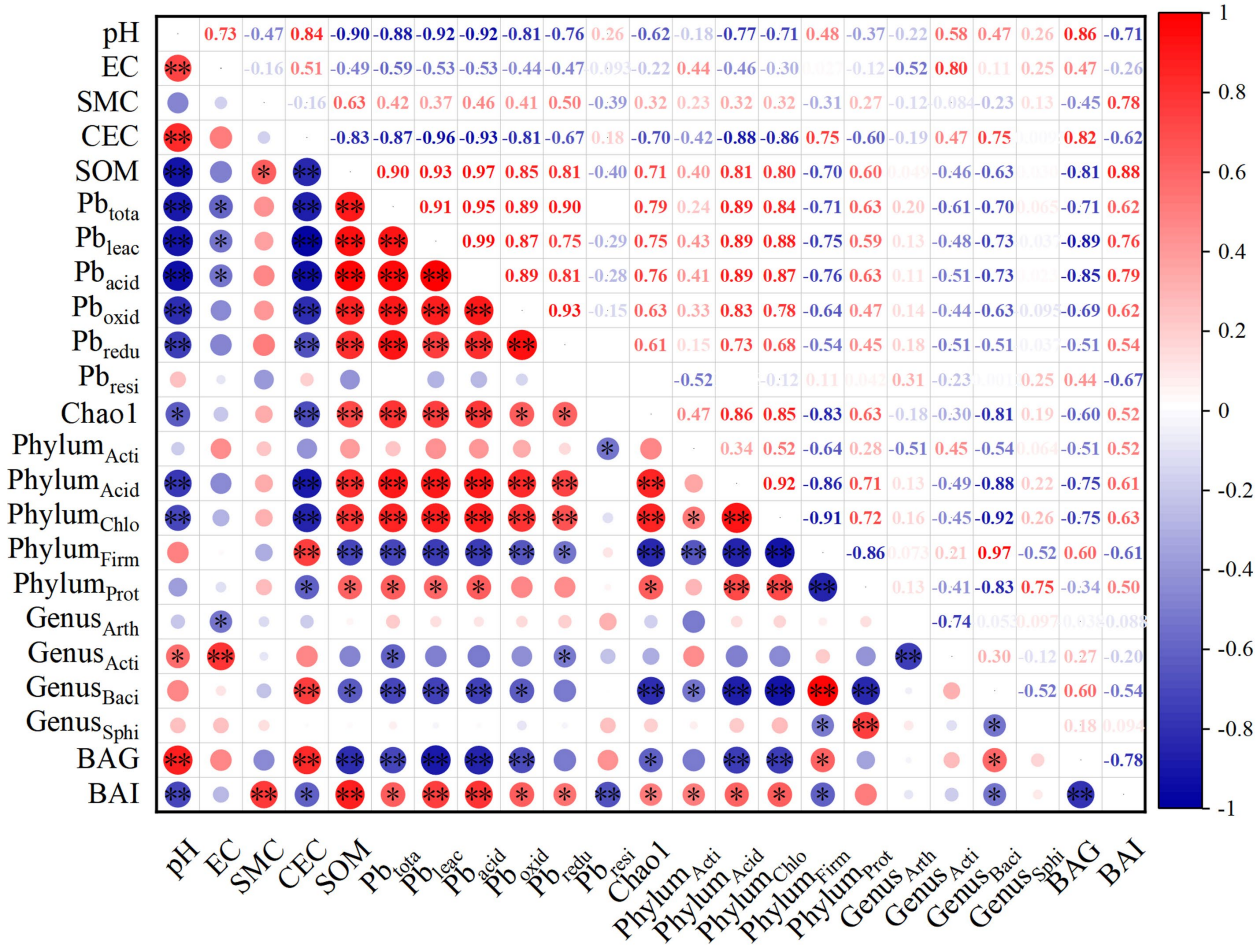
Heat map of Pb’s bioaccessibility correlation analysis. *represents the significance of Spearman’s correlation: *p ≤ 0.05, **p ≤ 0.01; Pb_tota_, Pb_leac_, Pb_acid_, Pb_oxid_, Pb_redu_, Pb_resi_ are total concentration, leachable concentration, acid-soluble, oxidation, reducible and residual states, respectively; Phylum_Acti_, Phylum_Acid_, Phylum_Chlo_, Phylum_Firm_, Phylum_Prot_ are Actinobacteriota, Acidobacteriota, Chloroflexi, Firmicutes and Proteobacteria, respectively; Genus_Arth_, Genus_Acti_, Genus_Baci_, Genus_Sphi_ are Arthrobacter, Actinotalea, Bacillus and Sphingomonas, respectively.

The analysis results indicate a strong correlation between soil physicochemical properties and Pb’s concentration (Figure 6). The main manifestation is that there is a significant positive correlation between pH, EC, CEC and the total concentration of Pb, leachate concentration, and effective state proportion; And a significant negative correlation between SMC, SOM and the total concentration of Pb, leachate concentration, and effective state proportion; But these variables have poor correlation with the residual state concentration of Pb. This indicates that the physicochemical properties of soil have a significant impact on the mobility and availability of Pb. The dissolution of salt in alkaline soil may increase the conductivity of soil solution or increase the negative charge on the surface of soil colloids, leading to stronger mobility and availability of Pb. Some studies had shown that soils with high SOM content often contain a large amount of amino and carboxyl groups, which can react with heavy metals in the soil through chelation and chelation, thereby reducing the migration ability of heavy metals (Gao et al., 2022).

#### 3.5.2 Correlation analysis between soil physicochemical properties, Pb concentration, and microbial community

Microbial communities participate in biogeochemical processes in soil and are closely related to soil physicochemical properties (Li et al., 2019; Chi et al., 2022). The analysis results indicate that pH and CEC are significantly negatively correlated with Acidobacteriota and Chloroflexi, and significantly positively correlated with Firmicutes (Figure 6). However, the correlation between soil physicochemical properties and other microbial species is not significant. Previous analysis has shown a significant correlation between soil physicochemical properties and Pb’s concentration. So we speculate that when soil is contaminated with heavy metals, the impact of soil physicochemical properties on microbial communities is indirectly reflected in changes in heavy metal content. Numerous studies had shown the impact of heavy metals on the abundance and community structure of microorganisms (Viti and Giovannetti, 2005; Garcia-Sanchez et al., 2015; Zhang et al., 2016). Due to the toxicity of heavy metals, they have a negative impact on microorganisms. However, the results of this study indicate a significant positive correlation between the total concentration of Pb and the Chao1 index (*R* = 0.79, **p ≤ 0.01). The effective concentration of Pb is significantly positively correlated with the species numbers of Acidobacteriota, Chloroflexi, and Proteobacteria, and significantly negatively correlated with the species numbers of Firmicutes, *Bacillus*, and *Actinotalia*. Some studies also indicated that there is no correlation between microorganisms and heavy metal’s concentration (Chen et al., 2014; Wang et al., 2021). We speculate that the analysis results may be related to extreme conditions of stabilization or F&T. Although stabilization treatment greatly reduces the total concentration and available state concentration of Pb, it may have a positive impact on the survival of microorganisms. However, the extreme conditions of F&T also significantly reduced the survival rate of most microorganisms, and creating favorable living conditions for tolerant species (Feng et al., 2007; Ou et al., 2019).

#### 3.5.3 Correlation analysis between various variables and bioaccessibility

Bioaccessibility is a key factor in determining the risks of contaminations to human health and developing effective remediation strategies (Juhasz et al., 2009; Lu et al., 2021). Many studies have analyzed the impact of different factors on Pb’s bioaccessibility, but there is still a lack of comprehensive research on the mechanisms and correlations between various influencing factors and the bioaccessibility of Pb in soil. Therefore, this section aims to discuss the correlation between soil physicochemical properties (pH, EC, SMC, CEC, SOM), Pb (leaching concentration, occurrence form), dominant phylum and genus of microbial species across different stages and bioaccessibility at the gastric and intestinal stages through Pearson correlation analysis (Figure 6).

##### 3.5.3.1 Correlation analysis between soil physicochemical properties and the bioaccessibility of Pb

PH, EC, and CEC showed a highly significant positive correlation (*p* ≤ 0.01), indicating that pH, EC, and CEC in soil are homologous. The analysis results indicated that the bioaccessibility of Pb decreases with increasing pH in the intestinal stage (Tang et al., 2006), while the opposite is true in the gastric stage. The change in soil pH generally affects the dissolution-precipitation, adsorption–desorption and other reactions in the soil, thereby affecting the bioaccessibility of heavy metals; SOM is significantly negatively correlated with the bioaccessibility of Pb in the gastric phase (*r* = −0.81, *p* ≤ 0.01), while it is significantly positively correlated with the intestinal phase (*r* = 0.88, *p* ≤ 0.01). Related studies had shown that the microbial community in intestinal fluid is relatively rich, and the reduction conditions formed by oxygen consumption are conducive to the desorption of heavy metals on the soil surface (Suriyagada et al., 2018); In addition, SMC is significantly positively correlated with the bioaccessibility of Pb in the intestinal stage (*r* = 0.78, *p* ≤ 0.01), indicating that as soil moisture content increases, the likelihood of lead intake in the intestinal stage increases.

##### 3.5.3.2 Correlation analysis between Pb’concentration and bioaccessibility

The analysis results indicate that Pb’s leaching concentration is negatively correlated with the bioaccessibility at stomach stage and positively correlated with the bioaccessibility at intestine stage. The effective state of heavy metals are sensitive to environmental changes, easy to migrate and release to be absorbed by the human body, and the potential risk to the human body is greater, while the chemical properties of the residue state are the most stable, it is difficult to migrate and transform, the effective state of lead is significantly positively correlated with the bioaccessibility of lead in the intestinal stage, and residue Pb is significantly negatively correlated with the bioaccessibility of Pb in the intestinal stage. However, acid-soluble state and oxidation state are significantly negatively correlated with the bioaccessibility of lead in the gastric stage, which is inconsistent with previous studies (Zhong et al., 2011).

##### 3.5.3.3 Correlation analysis between microbial community and bioaccessibility

Select microbial taxa at the phylum and genus levels with high abundance and significant changes in quantity across the three stages of soil for analysis. Among microorganisms at the phylum level, Acidobacteria and Chloroflexi, Actinobacteriota show a significant negative correlation with the bioaccessibility of Pb in the gastric stage, but a significant positive correlation in the intestinal stage. Firmicutes show the opposite trend. At the genus level, *Bacillus* shows a significant negative correlation with the bioavailability of Pb in the gastric phase, but a significant positive correlation in the intestinal phase.

##### 3.5.3.4 Analysis of the dominant factors causing changes in bioaccessibility

**Figure 7:**
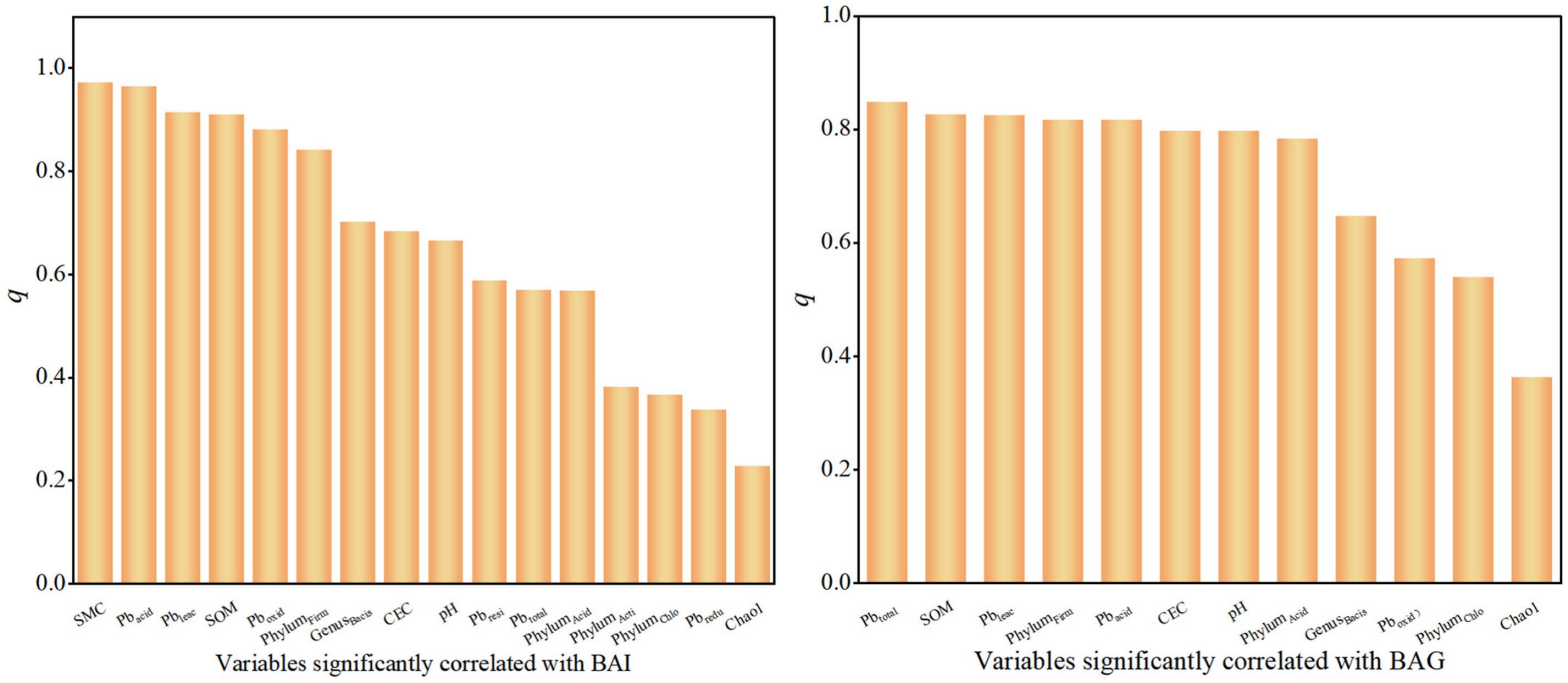
The explanatory power of factors.

To better interpret the impact of each variable on bioaccessibility, variables significantly correlated with BAG and BAI were selected for geographical detector analysis (Figure 7). The closer the *q* value (0 ~ 1) is to 1, the stronger the explanatory power of the variable for bioaccessibility. The results indicate that the total concentration of Pb has the greatest impact on BAG, with an explanatory power of 0.84. This suggests that the total concentration of Pb exerts a significant control over the variations in BAG and is a key factor influencing bioaccessibility in the gastric stage. Secondarily, factors such as soil organic matter and the leachate concentration of Pb are also important. Notably, Firmicutes and Acidobacteriota also exhibit strong explanatory power, with a value of approximately 0.8. Compared to BAG, the influence of each variable on the bioaccessibility in the intestinal stage is more distinct. The bioavailable forms of Pb (acid-soluble and oxidized states) have a significant impact on bioaccessibility in the intestinal stage. The soil texture (including SMC and SOM) also has an explanatory power of over 0.8 for the bioaccessibility in the intestinal stage. Overall, soil texture and Pb’s concentration in the soil are the primary controlling factors affecting bioaccessibility, while the impact of microbial community structure is relatively smaller.

## 4 Conclusion

In this study, the leaching, occurrence morphology, bioaccessibility and microbial community of lead in primitive, stabilized, stabilized and freeze–thaw soils are determined by horizontal oscillation, BCR three-step method, SBRC method and 16S rRNA high-throughput sequencing, and the following conclusions are obtained: (1) F&T cycles cause the alkalization and salinization of Pb-contaminated soil after stabilization treatment. F&T cycles reduce soil porosity and water-holding capacity, promote the mineralization of organic matter by microorganisms, and decrease the organic matter content in the soil. (2) The F&T cycles after stabilization will enhance the adsorption of Pb by soil particles and further reduce the mobility of Pb. (3) F&T cycles after stabilization, as well as the stabilization process itself, have little effect on the composition of microbial communities but significantly affected the community structure. Arthrobacter and Sphingomonas are more tolerant to Pb contamination, while *Bacillus* becomes the dominant genus after soil stabilization and F&T cycles, exhibiting strong resistance to environmental changes. (4) The bioaccessibility of Pb in the intestinal and gastric stages is significantly influenced by several soil properties, including pH, CEC, SOM, SMC, as well as the total content, leaching concentration, acid solubility, oxidation state, residue form of Pb, and the diversity of soil microbial species.

## Data Availability

The original contributions presented in the study are included in the article/supplementary material, further inquiries can be directed to the corresponding author.
